# Solid Lipid Nanoparticles of Myricitrin Have Antioxidant and Antidiabetic Effects on Streptozotocin-Nicotinamide-Induced Diabetic Model and Myotube Cell of Male Mouse

**DOI:** 10.1155/2018/7496936

**Published:** 2018-07-17

**Authors:** Akram Ahangarpour, Ali Akbar Oroojan, Layasadat Khorsandi, Maryam Kouchak, Mohammad Badavi

**Affiliations:** ^1^Department of Physiology, Faculty of Medicine, Diabetes Research Center, Health Research Institute, Ahvaz Jundishapur University of Medical Sciences, Ahvaz, Iran; ^2^Department of Physiology, Faculty of Medicine, Cellular and Molecular Research Center, Student Research Committee, Ahvaz Jundishapur University of Medical Sciences, Ahvaz, Iran; ^3^Department of Anatomical Sciences, Faculty of Medicine, Cellular and Molecular Research Center, Ahvaz Jundishapur University of Medical Sciences, Ahvaz, Iran; ^4^Department of Pharmaceutical Sciences, Faculty of Pharmacy, Nanotechnology Research Center, Ahvaz Jundishapur University of Medical Sciences, Ahvaz, Iran; ^5^Department of Physiology, Faculty of Medicine, Physiology Research Center, Ahvaz Jundishapur University of Medical Sciences, Ahvaz, Iran

## Abstract

Type 2 diabetes mellitus (T2DM) may occur via oxidative stress. Myricitrin is a plant-derived antioxidant, and its solid lipid nanoparticle (SLN) may be more potent. Hence, the present study was conducted to evaluate the effects of myricitrin SLN on streptozotocin-nicotinamide- (STZ-NA-) induced T2DM of the mouse and hyperglycemic myotube. In this experimental study, cold homogenization method was used to prepare SLN. Then, 120 adult male NMRI mice were divided into 7 groups: control, vehicle, diabetes (received STZ 65 mg/kg 15 min after injected NA 120 mg/kg), diabetes + SLN containing myricitrin 1, 3, and 10 mg/kg, and diabetes + metformin. For in vitro study, myoblast (C2C12) cell line was cultured and divided into 6 groups (*n* = 3): control, hyperglycemia, hyperglycemia + SLN containing myricitrin 1, 3, and, 10 *μ*M, and hyperglycemia + metformin. After the last nanoparticle treatment, plasma samples, pancreas and muscle tissues, and myotubes were taken for experimental assessments. Diabetes increased lipid peroxidation and reduced antioxidant defense along with the hyperglycemia, insulin resistance, and pancreas apoptosis. Hyperglycemia induced oxidative stress, antioxidant impairment, and cellular apoptosis. Myricitrin SLN improved diabetes and hyperglycemia complications in the in vivo and in vitro studies. Therefore, SLN of myricitrin showed antioxidant, antidiabetic, and antiapoptotic effects in the mouse and myotube cells.

## 1. Introduction

Diabetes mellitus is characterized by a group of metabolic disorders such as hyperglycemia over a long period, impaired carbohydrate, lipid, and protein metabolism caused by pancreas's failure to produce enough insulin, and insufficiency of the cells responding properly to the produced insulin. Type 2 diabetes mellitus (T2DM) is the most common form of diabetes mellitus, which making up to about 90% to 95% of all diabetes cases and is predicted to increase to 439 million by 2030. This is equivalent to about 6% of the world's population. It is estimated that this type of diabetes increase in the developing countries which more than 70% of the patients being 45–64 years old suffer from this disease [1, 2].

Glucose transporter type 4 (Glut-4), as a major insulin-stimulated glucose transporter, is expressed predominantly in skeletal muscle and facilitates the glucose transport into the skeletal muscle during insulin-stimulated glucose uptake. Glut-4 is sequestered in intracellular vesicles, and upon the plasma level of insulin increased during the postprandial period in response to the ingested carbohydrates and fat, Glut-4 vesicles translocate to and fuse with the plasma membrane, permitting increased glucose flux. Glut-4 is a rate-controlling transporter of glucose transport into muscle, and this disposal is diminished in insulin-resistant states. Also, a disruption in the normal insulin stimulated-glucose uptake into the skeletal muscle indicates a decrease or defect of Glut-4 expression and function caused by peripheral insulin resistance associated with T2DM [1–3]. Two main methods to evaluate glucose homeostasis in subjects at risk of diabetes are homeostasis model assessment of insulin resistance (HOMA-IR) and homeostasis model assessment of *β*-cell function (HOMA-*β*) that measuring quantifies insulin resistance and *β*-cell function, based on fasting insulin and glucose levels. These variables have been widely applied as an index of insulin resistance in individuals with both normal glucose tolerance and impaired glucose tolerance and have been used to document insulin resistance for patients at risk for T2DM [4, 5]. Metformin, belonging to the biguanide drug group, represents the first-line treatment of T2DM. This drug exerts its therapeutic effects via decreasing glucose production in the liver and reducing the intestinal absorption of glucose. These events lead to improving insulin sensitivity by increasing peripheral glucose uptake and utilization in muscles and other tissues. Metformin, along with antihyperglycemic properties, has several effects such as improvements in endothelial dysfunction, homeostasis and oxidative stress, insulin resistance, lipid profiles, and fat redistribution [6, 7].

There is a growing scientific evidence that oxidative stress and free radicals play an important role in the pathogenesis of T2DM and its complications through the induced damage in lipids, proteins, and nucleic acids. More recent studies indicated that oxidative stress could accelerate or contribute to the development of *β*-cell's dysfunction and insulin resistance. Hyperglycemia induces free radicals and impairs both enzymatic and nonenzymatic pathways of endogenous antioxidant defense. This condition can stimulate imbalance between the production and elimination of reactive oxygen species (ROS). Superoxide dismutase (SOD) and catalase (CAT) are enzymatic endogenous antioxidants that play synergistic actions in scavenging of free radicals by transforming them to less deleterious molecules. SOD catalyzes the dismutation from the superoxide anion (O_2_^−^), as a major ROS, into hydrogen peroxide (H_2_O_2_), and CAT converts H_2_O_2_ into H_2_O. Hence, a harmful free radical could be converting to the innocuous molecules. The main indicators of oxidative stress are polyunsaturated fatty acid peroxidation end products known as malondialdehyde (MDA). Higher levels of MDA and lower levels of antioxidant enzymes were observed in T2DM patients [8].

Bax and Bcl-2 are two proteins that regulate apoptosis in muscle cells. It was demonstrated that Bcl-2 expression could significantly elevate during hydrogen peroxide-induced apoptosis in myotubes and other muscle cells. Further, overexpression of Bcl-2 can decrease the level of proapoptotic Bax in myotubes, without significant changes in the levels of other proapoptotic agents such as Bak and Bad. It was generally approved that Bax and Bcl-2 serve to control the release of mitochondrial apoptotic factors in C2C12 myotubes following oxidative stress treatment. So, these genes are more important for apoptosis assessment than the others in the muscle cells [9].

Antioxidant therapy may inhibit the risk of developing diabetes and its complications in type 2 diabetic patients. A variety of antioxidants including plant-derived active substances interact with the free radicals through the binding of the metals which stimulate the production of free radicals, scavenge, or reduce their formation. These plants which contain antioxidant agents such as flavonoids have therapeutic effects on the treatment of T2DM [10]. Myricitrin (myricetin-3-O-*α*-rhamnoside), a botanical flavonol glycoside, belonging to the flavonol subgroup, was extracted from some plants (*Myrica rubra*, *Pouteria gender*, *Manilkara zapota*, and *Eugenia uniflora*). It has anxiolytic, antinociceptive, anti-inflammatory, and antioxidant effects [11]. Since this flavonoid glycoside has a high antioxidative activity, it is used as an important supplement in medicines. Further, myricitrin was shown to be a stronger free radical scavenger than other flavonol rhamnosides or quercetin. It was revealed that myricitrin inhibits ROS-induced vein endothelial cell dysfunction through the reducing of MDA, H_2_O_2_-induced oxidative damage, and regulation of antioxidant enzyme activity [12]. Also, the results of one study indicated that myricitrin showed higher hepatoprotective activity than silymarin and improved toxic liver damage via several mechanisms including antioxidant defense system preservation, inhibitors of inflammation, suppressors of profibrotic response, and enhancers of liver regeneration [13]. The bioavailability and metabolism of flavonoids, especially flavonoid glycoside, are the main properties to be considered. This compound is large and highly polar that cannot cross the membranes easily. Moreover, flavonoids have been extensive metabolism and hydrolysis by glycosidases in the cells of the liver, kidney, and gastrointestinal mucosa. So, these events may lead to reduce biological activity of myricitrin [14]. In recent researches, various nanoparticles have been created as carriers for drug and gene delivery such as solid lipid nanoparticles (SLNs). SLN may be a novel drug carrier system for oral delivery. This type of nanoparticle has several advantages, including high storage stability, reduces drug side effects concurrent with increase in bioavailability, and minimizes erratic absorption through its properties such as oral administration stability in the body fluids or plasma and decreased reticuloendothelial system uptake in the spleen and liver by induced hydrophilic molecule coating and altering surface characteristics [15].

The streptozotocin-nicotinamide (STZ-NA) model of T2DM is designed from the protective effects of NA against *β*-cell cytotoxic effects of STZ and was first introduced by Masiello et al. [16]. The severity of diabetic condition belongs to the administration of NA dosage in this model. It was revealed that 120 mg/kg of NA could induce a chronic hyperglycemia and its related metabolic disorders. This model of diabetes characterized by several properties such as stable hyperglycemia without exogenous insulin requirement reduced 40% of pancreatic *β*-cells and 60% of insulin stores, glucose intolerance, impaired glucose-stimulated insulin secretion, and the stability of metabolic alterations. STZ-NA model, as a nonobese model of T2DM, is reported to be more suitable for elucidation of the potential mechanisms of diabetic complications and biochemical or pharmacological assessment of antidiabetic drugs and natural compounds on the course of blood glucose-lowering and insulin-secretory properties and regenerative capacity of the endocrine pancreas [16, 17]. Also, this experimental model appears closer to human T2DM compare to other commonly used animal model [16]. Based on the previous considerations about the therapeutic effects of antioxidant component such as bioflavonoids on T2DM or hyperglycemic condition, and according to the low bioavailability of these agents, and since myotube cell is an important site of glucose utilization, the aim of this present study was conducted to evaluate the antidiabetic effect of SLN containing myricitrin on STZ-NA-induced diabetes and C2C12 cell of male mouse.

## 2. Materials and Methods

### 2.1. Reagents

The reagents are as follows: myricitrin (purity 98%) (AvaChem Scientific, USA), C2C12 (Pasteur Institute, Iran), Compritol®888 ATO (Sigma-Aldrich, France), oleic acid, propylene glycol, citrate buffer (citric acid monohydrate (C_6_H_8_O_7_·H_2_O)), trisodium citrate dihydrate (C_6_H_5_O_7_Na_3_·2H_2_O; pH 4.5), potassium hydroxide (KOH), sodium sulfate (Na_2_SO_4_), D-glucose, saline 0.9%, phenol, sulfuric acid (Merck, Germany), Tween 80 (Sinopharm Chemical Reagent Co. Ltd., China), phosphate-buffered saline (Pharmaceutical Technology Development Center of Ahvaz Jundishapur University of Medical Sciences, Iran; pH 7.4), proteinase K (Invitrogen, Thermo Fisher Scientific, Germany), fetal bovine serum (FBS), Dulbecco's modified eagle's medium (DMEM) (Solar Bio, South Korea), Trypsin-EDTA (Gibco, Canada), thiazolyl blue tetrazolium bromide (MTT), penicillin-streptomycin (Sigma-Aldrich, Canada), and dimethyl sulphoxide (DMSO) (Bio Idea, Iran).

### 2.2. Preparation of SLN

Since myricitrin is susceptible to the high temperature, cold homogenization method has been used in the present research. This method has been used to prepare SLNs loaded with myricitrin. Briefly, compritol was indirectly heated up to 65°C, then oleic acid as liquid lipid was added. The surfactant containing a mixture of Tween 80 and Span 20 (1 : 1) and myricitrin was dissolved in distilled water and added to the melt lipid phase. This mixture was sonicated at 90 W and 37°C for 2 min. The congelation was performed by adding a mixture of water/propylene glycol at 4°C (4 : 1) to reach 50 mL of volume, and this mixture was simultaneously homogenized using high-speed homogenizer (IKA® T25 digital ULTRA-TURRAX®, Germany) at 12000 ×g for 20 min.

### 2.3. Determination of Particle Size

After diluting SLN with myricitrin in double-distilled water, the particle size of nanoparticles was analyzed by Particle Size Analyzer instrument (Scatteroscope 1 Qudix, Korea). Each sample was assessed in triplicate (three-time readings were taken), and the average of the results was calculated.

### 2.4. Zeta Potential Assessment

The prepared myricitrin SLN zeta potential has been measured to determine the surface charge and stability of nanoparticles using the particle electrophoretic mobility procedure and the Zetasizer (Malvern Instruments Ltd., Worcestershire, UK) at 25°C. Myricitrin SLNs were dispersed in double-distilled water as dispersing medium (dispersant dielectric constant 80.0, viscosity 0.9781 cP, and dispersant refractive index 1.330). Each sample was measured in three-time readings.

### 2.5. Scanning Electron Microscopy (SEM) Imaging

Particle size and surface morphology of myricitrin SLNs were determined by SEM (Leo 1455 VP, Germany). Approximately 2 mg SLN containing myricitrin was dispersed in deionized water. A low amount of this suspension was dried on metal stub to form a thin uniform layer of nanoparticles. The metal stub was coated with 50 nm gold/palladium alloy. Finally, the gold-coated myricitrin SLNs were observed under SEM, and photographs were taken triplicate for each sample.

### 2.6. Determination of Encapsulation Efficiency and Encapsulation Capacity

Encapsulation efficiency (EE%) of myricitrin nanoparticles was determined by ultracentrifugation method. 2 mL of each sample was centrifuged (Vision Scientific Co. Ltd., Korea) 2 times at 30000 ×g for 25 min at 4°C to separate the SLN containing myricitrin from the unloaded drug. The amount of myricitrin in supernatant was determined by ultraviolet (UV) spectrophotometrical method (Analytik Jena AG, Germany) at 256 nm. The encapsulation efficiency was calculated by the following formula, which means that the amount of entrapped myricitrin was determined by subtracting the amount of unentrapped drug from the total amount of drug: EE% = (total drug − un − entrapped drug)/total drug × 100. The percentage of encapsulation capacity (EC%) was calculated by following formula: EC% = amount of drug loaded in lipid nanoparticles/weight of lipid (compritol) in SLN × 100 [18, 19].

### 2.7. In Vitro Release of Myricitrin

In vitro release of myricitrin was determined by dialysis membrane (12000 Dalton, Sigma-Aldrich, USA) method using ethanol and water (70–30%) (pH 7.4) as release medium. 1 mL of diluted myricitrin-loaded SLN suspensions with Tween 80 (3%) + normal saline (97%), as myricitrin solvent, containing 1 mg myricitrin was put into the dialysis membrane bags. The bags were suspended into 50 mL of distilled water, pH 7.4, and stirred at 80 ×g (37°C). At the intervals of 15, 30, and 45 min and 1, 2, 3, 4, 5, 6, 7, 8, and 24 h from the start of the experiment, 1 mL of dissolution medium was withdrawn and replaced by 1 mL of receptor medium. The concentration of released myricitrin was assessed by a UV spectrophotometer at 256 nm, and the cumulative percentage of drug release was calculated against time [20].

### 2.8. In Vivo Test

#### 2.8.1. Animals

In this experimental study, 120 adult (3-month-old) male NMRI mice weighing 25–30 g were obtained from the Ahvaz Jundishapur University of Medical Sciences (AJUMS) animal facility and were treated in accordance with the principles and guidelines on animal care of AJUMS as reviewed by an ethics committee (IR.AJUMS.REC.1395.136) and kept at a 20°C ± 4°C temperature with a 12-hour light/12-hour dark cycle. They had access to tap water and commercial chow ad libitum.

#### 2.8.2. Induction of Diabetes and Experimental Design

After one week of animal's acclimatization, for the sake of induce T2DM, a single dose of STZ (65 mg/kg) (Solar Bio, South Korea) dissolved in a citrate buffer (pH 4.5) was intraperitoneally injected 15 min after an intraperitoneal administration of NA (120 mg/kg) (Sigma-Aldrich, USA) dissolved in normal saline. Diabetes induction was confirmed by assaying blood glucose levels more than 200 mg/dL at 3 days after STZ-NA injection [21]. SLNs containing myricitrin, vehicle, and metformin (Alfa Aesar, Canada) (as a standard drug for T2DM treatment) were orally gavaged for 4 weeks in the treatment groups. So, animals were divided into 7 groups (*n* = 12): control, vehicle (injected one dose of STZ and NA solvent and gavaged Tween 80 (3%) + normal saline (97%) as myricitrin and its SLN solvent) [22], diabetes, diabetes + SLN containing myricitrin 1 mg/kg, diabetes + SLN containing myricitrin 3 mg/kg, diabetes + SLN containing myricitrin 10 mg/kg [23], and diabetes + metformin 200 mg/kg [24].

Since the half-life of myricitrin is about 12 h [25], 6 h after the last drug administration, the overnight fasted mice were anesthetized by ketamine/xylazine (70 mg/kg/10 mg/kg) (Alfasan, Netherlands), and the plasma samples were taken by cardiac puncture blood collection and centrifuging at 3500 ×g for 20 min. Then, the pancreas and muscle of all animals were immediately removed for histopathological and real-time PCR assessment, respectively. All plasma and tissue samples were kept at −80°C until experimental assessments were performed.

### 2.9. In Vitro Test

#### 2.9.1. Cell Culture

Skeletal myoblast (C2C12) cell line was purchased from the cellular bank of Pasteur Institute of Iran, Tehran, Iran. This cell was cultured in DMEM, supplemented with 10% FBS, 4 mM glutamine, penicillin-streptomycin (100 U/mL and 100 *μ*g/mL, resp.), under a humidified atmosphere of 5% CO_2_ at 37°C. The cells were cultured in 75 cm^2^ and divided into 25 cm^2^ cell culture flasks for SLN containing myricitrin treatment. The medium was replaced every 3 days until cultured cells arriving at 80% confluence [26, 27].

#### 2.9.2. Experimental Design

The grown C2C12 cells in 25 cm^2^ cell culture flasks were divided into 5 groups (*n* = 3): control (received medium containing D-glucose 5 mM), hyperglycemia (maintained in medium containing D-glucose 100 mM without any drug assessment), hyperglycemia + SLN containing myricitrin 1 *μ*M, hyperglycemia + SLN containing myricitrin 3 *μ*M, hyperglycemia + SLN containing myricitrin 10 *μ*M [28], and hyperglycemia + metformin 400 *μ*M [28]. The period of hyperglycemia only and concomitant with SLN of myricitrin administration was 48 h, and all administered flasks were incubated in the normal cell-cultured condition [29–31]. After the treating period, all cultured cells were collected by trypsin-EDTA (0.05%) and centrifuged at 1300 ×g for 15 min, resuspended in 0.5 mL of PBS (pH 7.4), and lysed using a Teflon homogenizer. All the samples were centrifuged at 2000 ×g for 10 min and kept at −80°C until experimental and real-time PCR measurements were performed [32].

### 2.10. Lipid Peroxidation and Antioxidant Enzyme Assessment

Pancreas tissue was removed, cut into small pieces on ice, and homogenized in 1/5 (*w/v*) PBS (pH 7.4) with a Teflon homogenizer. The supernatant of pancreas tissue and cultured cell homogenate were prepared via sample centrifuging at 2000 ×g for 10 min, and the levels of MDA, TAC, CAT (ZellBio, Germany), and SOD (Randox Laboratories Ltd., United Kingdom) were measured by specific commercial kits.

### 2.11. Diabetic Variable Measurement

At the end of experiment, fasting blood glucose was measured in overnight fasted mice by putting a drop of blood from the tip of the tail and using a digital glucometer (Elegance, Germany). A quantitative of plasma insulin level was performed by insulin ELISA kit (Monobind, USA). HOMA-IR and HOMA-*β* were carried out by homeostasis model assessment methods and were calculated using the following formula [33]:

(1) $$\begin{array}{c} HOMA‐IR=fasting\;insulin\left(µIU/mL\right)\times \frac{fasting\;glucose\left(mg/dL\right)}{405},\\ HOMA‐\beta =\frac{fasting\;insulin\left(µIU/mL\right)\times 360}{fasting\;glucose\left(mg/dL\right)–63}. \end{array}$$

### 2.12. Glycogen Content of Skeletal Muscle

For muscle glycogen content measurement, 100 mg of mouse soleus and gastrocnemius muscles and C2C12 cells cultured in 25 cm^2^ flasks were removed, and 0.5 mL of 30% KOH solution saturated with Na_2_SO_4_ was added to the sample tubes, and the samples were heated at 100°C for 30 min and incubated on ice and added 1.2 volume of 95% ethanol to the tubes. All samples stood on ice for 30 min before centrifugation at 840 ×g for 30 min. The supernatants were removed, and the glycogen precipitates were resuspended in (1 mL for skeletal muscle and 50 *μ*L for C2C12 cells) distilled water. Then, 1 mL for skeletal muscle and 50 *μ*L for C2C12 cells of 5% phenol solution were added to the aliquots of glycogen solution, and 5 mL for skeletal muscle and 250 *μ*L for C2C12 cells of 98% sulfuric acid were rapidly added, and the absorbance was measured with a plate reader at 490 nm. The glycogen content in the muscle and C2C12 cells was calculated from the standard curve of D-glucose and expressed as mg/g wet muscle weight and percent of control, respectively [34].

### 2.13. MTT Assay Experiment

The concentration of MTT 5 mg/mL was prepared in PBS to determine the cellular viability. Live cells convert the MTT compound to the formed formazan crystals. These crystals were dissolved by DMSO. So, removed cultured cells were incubated in medium containing 0.5 mg/mL of 3-(4,5-dimethylthiazol-2-yl)-2,5-diphenyltetrazolium bromide for 4 h (at 5% CO_2_ and 37°C). The purple formazan crystals were dissolved in 100 *μ*L DMSO and shacked for 15 min, and the absorbance MTT assessment was read at 540 nm using an ELISA reader (ELx808 Absorbance Microplate Reader, ELISA Technologies Inc., USA) [35, 36].

### 2.14. Glut-4 and Apoptotic Gene Expression Assessment

Total RNA was purified from the muscle and C2C12 cells using the commercial instruction of RNeasy mini kit (Qiagen, Valencia, CA). cDNA was synthesized using Reverse Transcriptase kit (Takara, Japan) according to the manufacturer's instructions. Real-time PCR was performed with SYBR green Master Mix (Takara, Japan) in ABI StepOnePlus instrument (Thermo Fisher, USA) and was prepared in duplicate. The protocol of real-time PCR was conducted under the following conditions: 2 min at 60°C and 10 min at 95°C, followed by 40 cycles at 95°C for 15 seconds and 60°C for 60 seconds, 1 cycle of 95°C for 15 seconds, 60°C for 30 seconds, and 95°C for 15 seconds. The relative Glut-4, Bax, and Bcl-2 gene expression levels to the expression level of GAPDH, as the endogenous reference gene, were calculated using a comparative 2-delta delta cycle threshold (2^−ΔΔCT^) method (2^−ΔΔCT^ = [(Ct Glut-4 of treated mice − Ct GAPDH of treated mice) − (Ct Glut-4 of untreated mice − Ct GAPDH of untreated mice)]). Before using this method for quantitation, a primer efficiency investigation was conducted to validate our experiment. The primer sequences of the forward and reverse regarding Glut-4, Bax, Bcl-2, and GAPDH gene (Microsynth, Switzerland) were [27] as follows:

Glut-4 forward primer, 5′-CGCACTAGCTGAGCTGAAGG-3′ and Glut-4 reverse primer, 5′-GCAGCACCACTGCGATGATA-3′; Bax forward primer, 5′-GCTGGACATTGGACTTCCTC-3′ and Bax reverse primer, 5′-ACCACTGTGACCTGCTCCA-3′; Bcl-2 forward primer, 5′-GCTGGACATTGGACTTCCTC-3′ and Bcl-2 reverse primer, 5′-GCTGGACATTGGACTTCCTC-3′; and GAPDH forward primer, 5′-ACCCAGAAGACTGTGGATGG-3′ and GAPDH reverse primer, 5′-TTCTAGACGGCAGGTCAGGT-3′.

### 2.15. Histological Assessment of Pancreas Tissue

The pancreas tissue samples of animals were fixed in formalin solution (10%). All tissues were embedded in paraffin after dehydrated in graded alcohol concentrations. Pancreatic sections of 5–7 *μ*m were prepared and stained with hematoxylin and eosin (H&E) (Sigma-Aldrich, USA). Eight microscopic stained slides per mouse were examined for the assessment of islet diameters. This measurement was analyzed using Motic Images Plus 2.0 image analysis software. The slide reading was conducted under a blind method [37].

### 2.16. Pancreatic Tissue Apoptosis Assessment

TUNEL staining was carried out based on labeling of the DNA strand breaks by the In Situ Cell Death Detection Kit, POD (Roche Applied Science, Germany). The paraffin sections of pancreas tissue were dewaxed and incubated with Proteinase K for 0.5 h at 24°C. After washing with PBS, the sections were incubated with TUNEL reaction mixture in a humidity chamber at 37°C for 1 h. The sections were incubated with antifluorescein-AP for 30 min at 37°C after washing with PBS. The slides were washed in deionized water and incubated with DAB substrate for 5 min. Intense, dark brown stained nucleus was considered as TUNEL-positive cells. Percentage of TUNEL-positive cells (apoptotic index) was calculated randomly in 10 fields for each slide. Three slides per animal were used for this method [38].

### 2.17. Statistical Assessment

The results were statistically analyzed using SPSS software (version 16) with one-way analysis of variance (ANOVA), followed by post hoc least significant difference (LSD) tests. Further, data were represented as mean ± standard error (SE) and differences were considered statistically significant at *p* < 0.05.

## 3. Results

### 3.1. Characterization of Myricitrin SLNs

**Figure 1:**
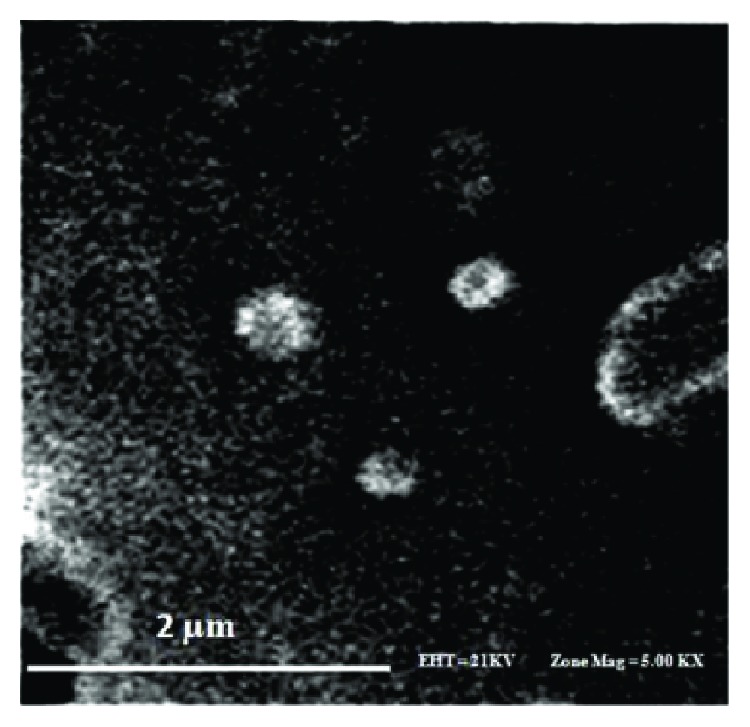
Scanning electron microscope (SEM) image of myricitrin SLNs.

**Figure 2:**
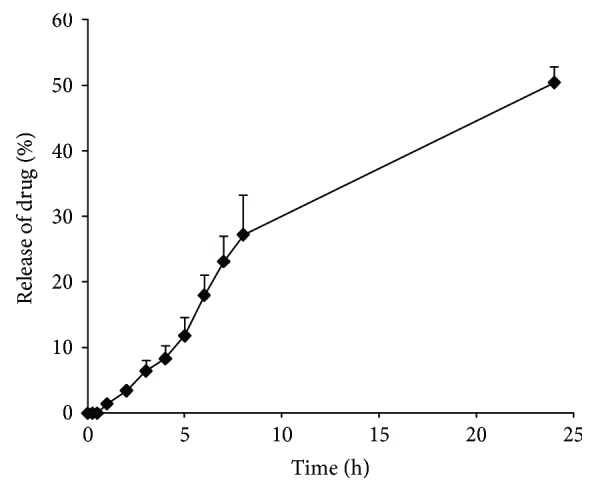
In vitro release of myricitrin from its SLN.

The mean particle size and zeta potential were 76.1 nm and− 5.51 mV, respectively. Encapsulation efficiency (EE) and encapsulation capacity (EC) were 56.2% and 5.62%, respectively. The nanoparticles showed spherical shapes under SEM, and the particle size ranged from 50 to 150 nm (Figure 1). The release of myricitrin from SLNs occurred in two phases. The release of myricitrin from its SLNs did not occur at the intervals of 15, 30, and 45 min, because the lipid skeleton of the SLN as part of the drug was absorbing water. Next, the release of myricitrin from its SLNs was 1.435, 3.435, 6.432, 8.318, 11.789, 17.972, 23.1, 27.184, and 50.339% at 1, 2, 3, 4, 5, 6, 7, 8, and 24 h, respectively (Figure 2).

### 3.2. Role of SLN Containing Myricitrin on Body and Tissue Weight

**Figure 3:**
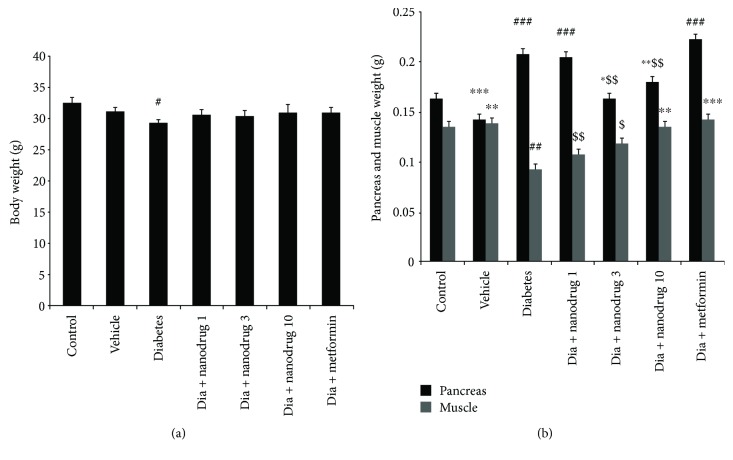
Effect of myricitrin SLN on body and tissue weight. Data are presented as mean ± SE; *n* = 12; ^#^*p* < 0.05, ^##^*p* < 0.01, and ^###^*p* < 0.001 compared with control; ^∗^*p* < 0.05, ^∗∗^*p* < 0.01, and ^∗∗∗^*p* < 0.001 compared with diabetes; ^$^*p* < 0.05, ^$$^*p* < 0.01, and ^$$$^*p* < 0.001 compared with diabetes + metformin (one-way analysis of variance (ANOVA), followed by post hoc least significant difference (LSD) tests). (a) Body weight; (b) tissue weight.

The body weight reduced in the untreated diabetic group compared to the control (*p* < 0.05) (Figure 3(a)). The pancreas weight increased in diabetes, diabetic SLN of myricitrin 1 mg/kg, and metformin-administered mice compared to control (*p* < 0.001). This variable decreased in vehicle (*p* < 0.001), diabetes + SLN containing myricitrin 3 mg/kg (*p* < 0.05), and 10 mg/kg (*p* < 0.01) when compared to the diabetes group. Further, the weight of the pancreas in diabetes + SLN containing myricitrin 3 (*p* < 0.01) and 10 mg/kg (*p* < 0.001) showed a significant decrease versus the metformin group. The weight of mouse muscle showed a significant decrease in the untreated diabetic group when compared to the control group (*p* < 0.01). This variable increased in the vehicle (*p* < 0.01), diabetes + SLN containing myricitrin 10 mg/kg (*p* < 0.01), and diabetes + metformin groups (*p* < 0.001) compared with the diabetes group. Also, there was a significant muscle weight reduction in the diabetes + SLN containing myricitrin 1 (*p* < 0.01) and 3 mg/kg (*p* < 0.05) compared to diabetes + metformin (Figure 3(b)).

### 3.3. Malondialdehyde and Antioxidant Enzyme Level

**Figure 4:**
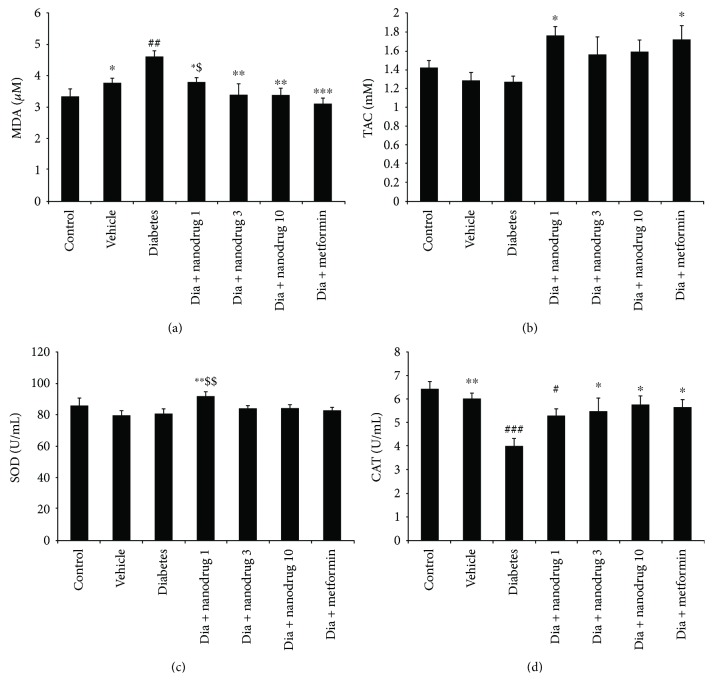
Effect of myricitrin SLN on MDA, TAC, and antioxidant enzyme level of the pancreas. Data are presented as mean ± SE; *n* = 12; ^#^*p* < 0.05, ^##^*p* < 0.01, and ^###^*p* < 0.001 compared with control; ^∗^*p* < 0.05, ^∗∗^*p* < 0.01, and ^∗∗∗^*p* < 0.001 compared with diabetes; ^$^*p* < 0.05 and ^$$^*p* < 0.01 compared with diabetes + metformin (one-way analysis of variance (ANOVA), followed by post hoc least significant difference (LSD) tests). (a) MDA, (b) TAC, (c) SOD, and (d) CAT.

**Figure 5:**
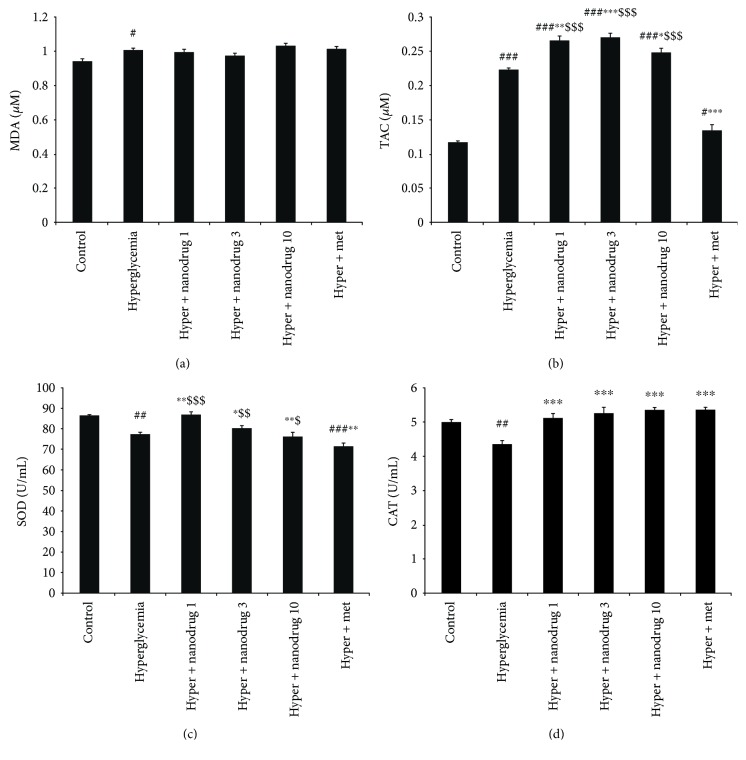
Effect of myricitrin SLN on MDA, TAC, and antioxidant enzyme level of C2C12 cell. Data are presented as mean ± SE; *n* = 3; ^#^*p* < 0.05, ^##^*p <* 0.01, and ^###^*p <* 0.001 compared with control; ^∗^*p* < 0.05, ^∗∗^*p* < 0.01, and ^∗∗∗^*p* < 0.001 compared with hyperglycemia; ^$^*p* < 0.05, ^$$^*p* < 0.01, and ^$$$^*p* < 0.001 compared with hyperglycemia + metformin (one-way analysis of variance (ANOVA), followed by post hoc least significant difference (LSD) tests). (a) MDA, (b) TAC, (c) SOD, and (d) CAT.

Pancreatic level of malondialdehyde increased in the diabetes group (*p* < 0.01) compared to the control. Also, compared with the diabetes group, this lipid peroxidation marker decreased in the vehicle (*p* < 0.05), diabetes + SLN containing myricitrin 1 (*p* < 0.05), 3 (*p* < 0.01), and 10 mg/kg (*p* < 0.01), and diabetes + metformin groups (*p* < 0.001). Metformin administered in diabetic mice reduced more the level of MDA than SLN containing myricitrin 1 mg/kg (*p* < 0.05) (Figure 4(a)). The results of total antioxidant capacity (TAC) showed a remarkable increase in the diabetes + SLN containing myricitrin 1 mg/kg and diabetes + metformin groups (*p* < 0.05) compared with the diabetes group (Figure 4(b)). Pancreatic level of SOD increased in the diabetes + SLN containing myricitrin 1 mg/kg group versus the untreated diabetic mice and diabetes + metformin groups (*p* < 0.01) (Figure 4(c)). CAT measurement revealed a significant decrease in the diabetes (*p* < 0.001) and diabetes + SLN containing myricitrin 1 mg/kg (*p* < 0.05) groups when compared to the control. Further, this enzyme level increased in the vehicle (*p* < 0.01), diabetes + SLN containing myricitrin 3 and 10 mg/kg and diabetes + metformin groups (*p* < 0.05) compared to the diabetes group (Figure 4(d)). In vitro results indicated that a significant increase of MDA has occurred in the hyperglycemia group compared to the control (*p* < 0.05) (Figure 5(a)). TAC assessment showed an increased in all groups with *p* < 0.001 and hyperglycemia + metformin with *p* < 0.05 compared with the control. This variable increased in the hyperglycemia + SLN containing myricitrin 1 (*p* < 0.01), 3 (*p* < 0.001), and 10 *μ*M (*p* < 0.05) groups and decreased in the hyperglycemia + metformin group (*p* < 0.001) compared to the hyperglycemia group. TAC increased significantly in the hyperglycemia + SLN containing 1, 3, and 10 *μ*M groups (*p* < 0.001) versus the hyperglycemia + metformin group (Figure 5(b)). SOD enzyme level in myotubes reduced in the hyperglycemia (*p* < 0.01), hyperglycemia + SLN containing 3 (*p* < 0.05) and 10 *μ*M (*p* < 0.01), and hyperglycemia + metformin groups (*p* < 0.001) compared to the control. This antioxidant enzyme increased in the hyperglycemia + SLN containing myricitrin 1 *μ*M (*p* < 0.01) and decreased in hyperglycemia + metformin (*p* < 0.01) groups versus the untreated hyperglycemic exposed myotubes. The results of metformin administration showed a significant decrease in SOD when compared to the hyperglycemia + SLN containing myricitrin 1, 3, and 10 *μ*M groups (*p* < 0.001, *p* < 0.01, and *p* < 0.05, resp.) (Figure 5(c)). CAT assessment demonstrated a remarkable reduction in the hyperglycemia group compared to the control group (*p* < 0.01). Further, a significant increase was observed in all treated groups when compared with the hyperglycemia group (*p* < 0.001) (Figure 5(d)).

### 3.4. Antidiabetic Effects of Myricitrin SLNs

**Figure 6:**
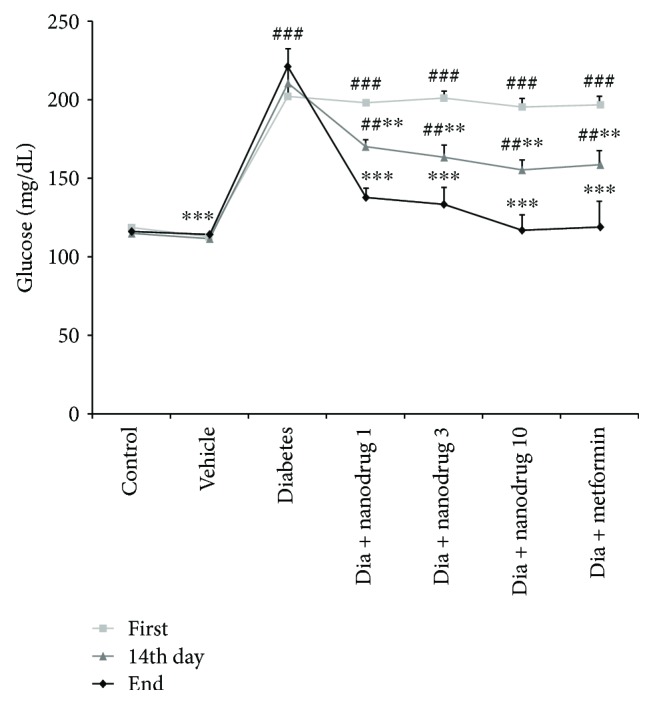
Effect of myricitrin SLN on blood glucose level at the first day, 14th day, and end of experimental period. Data are presented as mean ± SE; *n* = 12; ^##^*p* < 0.01 and ^###^*p* < 0.001 compared with control; ^∗∗^*p* < 0.01 and ^∗∗∗^*p* < 0.001 compared with diabetes (one-way analysis of variance (ANOVA), followed by post hoc least significant difference (LSD) tests).

The blood glucose level at the first day of experiment increased in all groups except the vehicle group compared to the control group (*p* < 0.001). This variable increased in all treated groups compared to the control and increased in that groups versus the diabetes group at the fourteenth day of the experiment (*p* < 0.01). The blood glucose level increased in the diabetes group compared to the control and decreased in the vehicle group and all drug-administered mice versus the diabetes group at the end of experimental period (*p* < 0.001) (Figure 6). Insulin measurement showed a significant increase in the diabetes (*p* < 0.05), diabetes + SLN containing myricitrin, and diabetes + metformin-administered mice (*p* < 0.001) when compared to the control. Further, this hormone level decreased in the vehicle (*p* < 0.05) and increased in the diabetes + SLN containing myricitrin 3 (*p* < 0.05) and 10 mg/kg (*p* < 0.01) and diabetes + metformin groups (*p* < 0.001) compared to the diabetes group. Plasma level of insulin decreased in mice administered with diabetic SLN containing myricitrin 1, 3, and 10 mg/kg when compared to the diabetes + metformin group (*p* < 0.01, *p* < 0.01, and *p* < 0.05, resp.). HOMA-IR increased in the diabetes, diabetes + SLN containing myricitrin, and diabetes + metformin-treated mice when compared to the control (*p* < 0.001). This insulin resistance index reduced in the vehicle (*p* < 0.001), diabetes + SLN containing myricitrin 1, 3, (*p* < 0.05) and 10 mg/kg- (*p* < 0.01) administered animals versus the diabetes group. HOMA-*β* decreased in the diabetes (*p* < 0.001) and increased in diabetes + SLN containing myricitrin 1 (*p* < 0.05), 3, and 10 mg/kg, and diabetes + metformin (*p* < 0.001) groups compared with the control. Furthermore, the assessment of the vehicle, diabetes + SLN containing all dose of myricitrin, and diabetes + metformin groups revealed a significant increase in this variable when compared to the untreated diabetic mice (*p* < 0.001). This *β*-cell function index showed a significant decrease in the diabetes + SLN containing myricitrin 1, 3, and 10 mg/kg in comparison with the diabetes + metformin group (*p* < 0.001, *p* < 0.001, and *p* < 0.01, resp.) (Table 1).

### 3.5. Glycogen Content of Skeletal Muscle and C2C12

**Figure 7:**
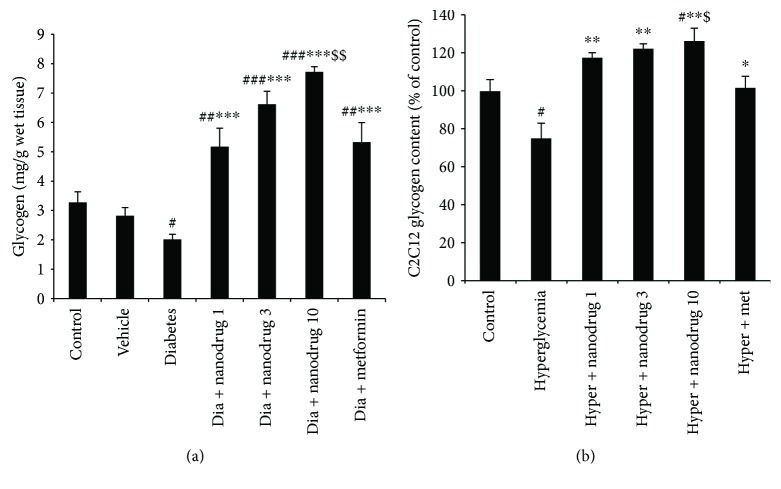
Effect of myricitrin SLN on glycogen content of skeletal muscle and C2C12 cell. Data are presented as mean ± SE; *n* = 12 for skeletal muscle and *n* = 3 for C2C12 cell; ^#^*p* < 0.05, ^##^*p* < 0.01, and ^###^*p* < 0.001 compared with control; ^∗^*p* < 0.05, ^∗∗^*p* < 0.01, and ^∗∗∗^*p* < 0.001 compared with diabetes or hyperglycemia; ^$^*p* < 0.05 and ^$$^*p* < 0.01 compared with diabetes + metformin or hyperglycemia + metformin (one-way analysis of variance (ANOVA), followed by post hoc least significant difference (LSD) tests). (a) Glycogen content in skeletal muscle; (b) glycogen content in C2C12 cell.

As shown in Figure 7(a), muscle's glycogen decreased in the diabetes (*p* < 0.05) and increased in diabetic SLN 1 (*p* < 0.01), 3, and 10 mg/kg (*p* < 0.001) and metformin- (*p* < 0.01) treated groups when compared to the control. Also, SLN containing myricitrin 1, 3, and 10 mg/kg and metformin administration increased the storage of muscle's glycogen compared with the diabetes group (*p* < 0.001). SLN containing myricitrin 10 mg/kg in diabetic mice indicated a significant increase in this variable versus the diabetes + metformin group (*p* < 0.01). C2C12 glycogen content measurement revealed a significant decrease in untreated hyperglycemic cells when compared to the control (*p* < 0.05). Further, this variable increased in the hyperglycemia + SLN containing myricitrin 10 *μ*M- (*p* < 0.05) treated groups compared with the control. Myotube glycogen content increased in the hyperglycemia + SLN containing myricitrin 1, 3, and 10 *μ*M (*p* < 0.01) and hyperglycemia + metformin groups (*p* < 0.05) versus the untreated hyperglycemic cells. Also, SLN containing myricitrin 10 *μ*M (*p* < 0.05) administration increased the content of glycogen in hyperglycemic C2C12 cells compared to metformin (Figure 7(b)).

### 3.6. Glut-4 Gene Expression

**Figure 8:**
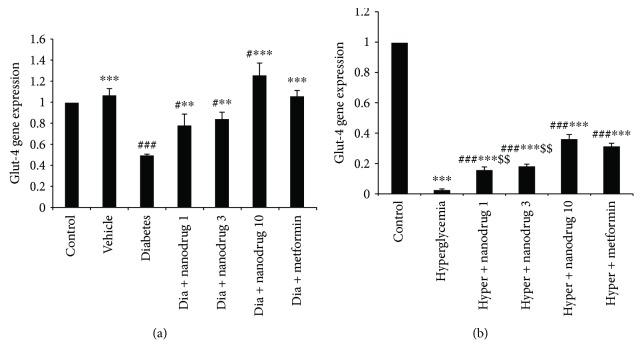
Glut-4 gene expression in skeletal muscle and C2C12 cell. Data are presented as mean ± SE; *n* = 12 for skeletal muscle and *n* = 3 for C2C12 cell; ^#^*p* < 0.05 and ^###^*p* < 0.001 compared with control; ^∗∗^*p* < 0.01 and ^∗∗∗^*p* < 0.001 compared with diabetes or hyperglycemia; ^$$^*p* < 0.01 compared with diabetes + metformin or hyperglycemia + metformin (one-way analysis of variance (ANOVA), followed by post hoc least significant difference (LSD) tests). (a) Glut-4 gene expression in skeletal muscle; (b) Glut-4 gene expression in C2C12 cell.

The results of gene assessment indicated that muscle's Glut-4 gene expression significantly decreased in the diabetes (*p* < 0.001) and diabetes + SLN containing myricitrin 1 and 3 mg/kg groups and increased in the diabetes + SLN containing myricitrin 10 mg/kg compared to the control (*p* < 0.05). This variable increased in the vehicle (*p* < 0.001), diabetes + SLN containing myricitrin 1, 3 (*p* < 0.01), and 10 mg/kg, and diabetes + metformin groups (*p* < 0.001) when compared with the untreated diabetic mice (Figure 8(a)). Glut-4 gene expression was significantly reduced in all treated C2C12 cell groups compared to the control (*p* < 0.001). This gene expression increased in all treated groups when compared to the hyperglycemia group (*p* < 0.001). The comparison between metformin and SLN administration revealed a significant increase of this gene expression in the hyperglycemia + metformin group versus the hyperglycemia + SLN containing myricitrin 1 (*p* < 0.001) and 3 *μ*M groups (*p* < 0.001) (Figure 8(b)).

### 3.7. Cellular Viability

**Figure 9:**
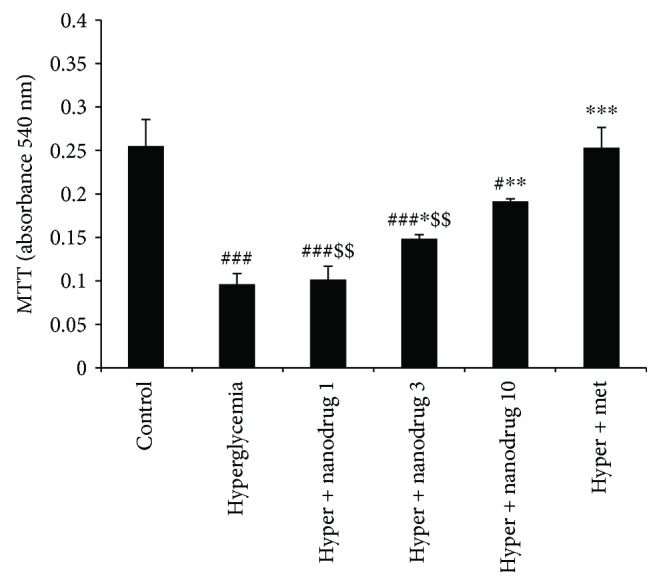
C2C12 cell survival. Data are presented as mean ± SE; *n* = 5; ^#^*p* < 0.05 and ^###^*p* < 0.001 compared with control; ^∗^*p* < 0.05, ^∗∗^*p* < 0.01, and ^∗∗∗^*p* < 0.001 compared with hyperglycemia; ^$$^*p* < 0.01 compared with hyperglycemia + metformin (one-way analysis of variance (ANOVA), followed by post hoc least significant difference (LSD) tests).

The results of MTT assessment demonstrated that this variable decreased in the hyperglycemia, hyperglycemia + SLN containing myricitrin 1, 3 (*p* < 0.001), and 10 *μ*M groups (*p* < 0.05) compared to the control. This variable revealed an increase in SLN containing myricitrin 3 (*p* < 0.05), 10 *μ*M (*p* < 0.01), and hyperglycemia + metformin (*p* < 0.001) groups when compared to the untreated hyperglycemic C2C12 cells. Further, metformin treatment showed a significant increase in cellular viability compared with hyperglycemia + SLN containing myricitrin 1 and 3 *μ*M groups (*p* < 0.01) (Figure 9).

### 3.8. Myotube Cell Apoptotic Gene Expression

**Figure 10:**
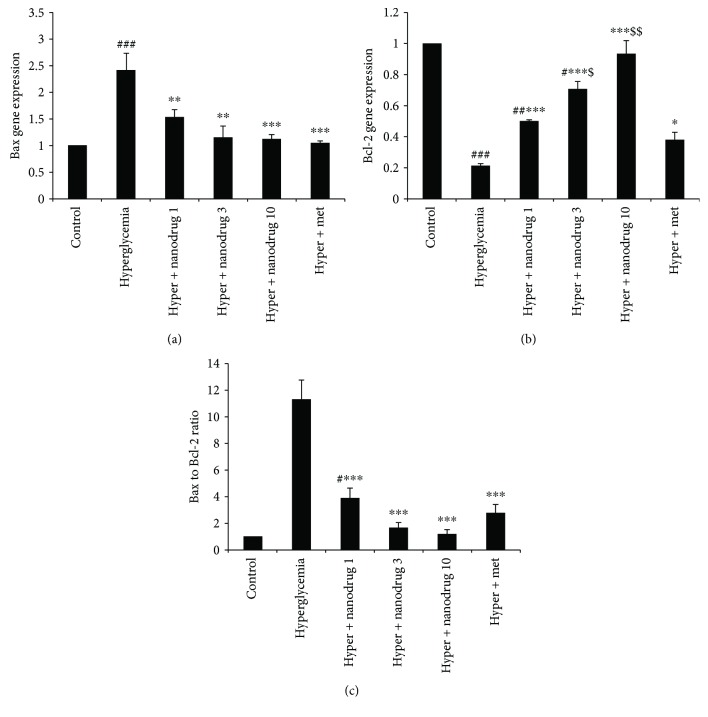
Effect of myricitrin SLN on C2C12 apoptosis gene expression. Data are presented as mean ± SE; *n* = 3; ^#^*p* < 0.05, ^##^*p* < 0.01, and ^###^*p* < 0.001 compared with control; ^∗^*p* < 0.05, ^∗∗^*p* < 0.01, and ^∗∗∗^*p* < 0.001 compared with hyperglycemia; ^$^*p* < 0.05 and ^$$^*p* < 0.01 compared with hyperglycemia + metformin (one-way analysis of variance (ANOVA), followed by post hoc least significant difference (LSD) tests).

The level of Bax gene expression revealed a significant increase in the hyperglycemia group compared to the control (*p* < 0.001). This variable decreased in the hyperglycemia + SLN containing myricitrin 1, 3 (*p* < 0.01), and 10 μM and hyperglycemia + metformin (*p* < 0.001) groups versus the hyperglycemia group (Figure 10(a)). Bcl-2 assessment showed a remarkable decrease in the hyperglycemia (*p* < 0.001), hyperglycemia + SLN containing myricitrin 1 (*p* < 0.01) and 3 *μ*M (*p* < 0.05) groups when compared to the control. This gene expression increased in the SLN containing all doses of myricitrin (*p* < 0.001) and metformin- (*p* < 0.05) treated groups versus the hyperglycemia group. Further, there were significant differences between metformin and SLN containing myricitrin 3 (*p* < 0.05) and 10 *μ*M (*p* < 0.01) administration in Bcl-2 levels (Figure 10(b)). Bax to Bcl-2 ratio level showed an enhancement in the hyperglycemia (*p* < 0.001) and hyperglycemia + SLN containing myricitrin 1 *μ*M (*p* < 0.05) groups compared to the control. Also, this ratio decreased in all treated groups compared with the hyperglycemia group (*p* < 0.001) (Figure 10(c)).

### 3.9. Histological Changes of Pancreas Tissue

**Figure 11:**
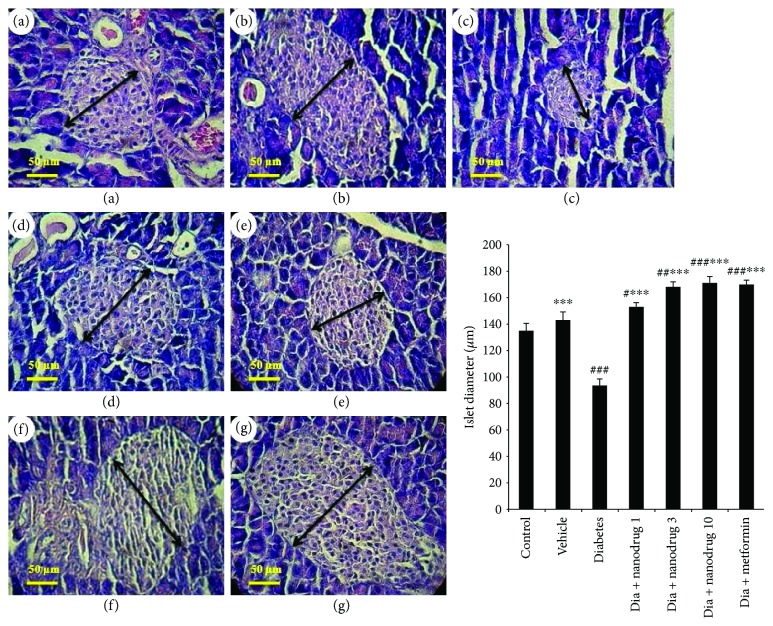
Role of myricitrin SLN on islet diameter (hematoxylin and eosin; ×40 magnification). Data are presented as mean ± SE; *n* = 8; ^#^*p* < 0.05, ^##^*p* < 0.01, and ^###^*p* < 0.001 compared with control; ^∗∗∗^*p* < 0.001 compared with diabetes (one-way analysis of variance (ANOVA), followed by post hoc least significant difference (LSD) tests). (a) Control; (b) vehicle; (c) diabetes; (d) diabetes + SLN containing myricitrin 1 mg/kg; (e) diabetes + SLN containing myricitrin 3 mg/kg; (f) diabetes + SLN containing myricitrin 10 mg/kg; (g) diabetes + metformin.

As demonstrated in Figure 11, the islet diameter decreased in diabetes (*p* < 0.001) and increased in diabetes + metformin and diabetes + SLN containing myricitrin 1 (*p* < 0.05), 3 (*p* < 0.01), and 10 mg/kg (*p* < 0.001) groups compared to the control. SLN containing myricitrin 1, 3, and 10 mg/kg and metformin (*p* < 0.001) administered in diabetic animals improved this diameter reducing when compared to the diabetes group. Similar effect was observed in the vehicle group compared to the diabetes group (*p* < 0.001).

### 3.10. Role of Myricitrin SLNs on Pancreatic Apoptosis

**Figure 12:**
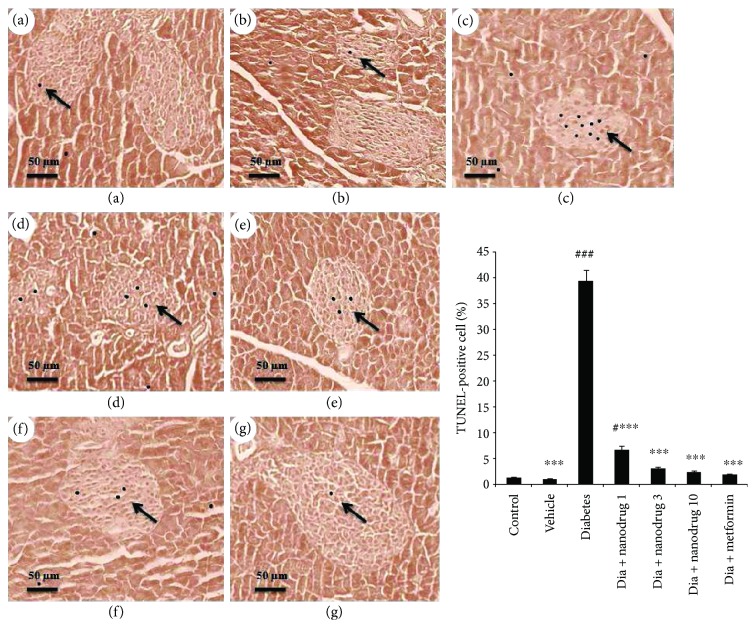
Effect of myricitrin SLN on pancreas apoptosis (TUNEL) (×40 magnification). Data are presented as mean ± SE; *n* = 3; ^#^*p* < 0.05 and ^###^*p* < 0.001 compared with control; ^∗∗∗^*p* < 0.001 compared with diabetes (one-way analysis of variance (ANOVA), followed by post hoc least significant difference (LSD) tests). (a) Control; (b) vehicle; (c) diabetes; (d) diabetes + SLN containing myricitrin 1 mg/kg; (e) diabetes + SLN containing myricitrin 3 mg/kg; (f) diabetes + SLN containing myricitrin 10 mg/kg; (g) diabetes + metformin.

Pancreatic cell apoptosis was significantly increased in the diabetes (*p* < 0.001) and diabetes + SLN containing myricitrin 1 mg/kg groups (*p* < 0.05) compared with the control group. This variable improved in all diabetic treated mice when compared to the diabetes group (*p* < 0.001) (Figure 12).

## 4. Discussion

Some of the nanoparticle advantages are drug protection from the rapid metabolism and nonspecific recognition or distribution. It does appear that two important variables which affect the cellular nanoparticle uptake are the size and shape. The cellular uptake of nanoparticles (100 nm) was 2.5 and 6 times more than microparticles (1 *μ*m and 10 *μ*m), respectively. Nanoparticles less than 10 nm were eliminated by renal rapidly, and if the diameter arrived at 200 nm, the vascular permeability decreases; hence, it is quickly removed by the activation of a complementary system in the bloodstream. It was revealed that the spherical nanoparticle uptake is 5 times greater than rod-shaped particles. The nanoparticle surface properties such as hydrophobicity and hydrophilicity are important for blood component absorbance including opsonins. Surface charge is the main factor for surface properties because less opsonization occurs in neutrally charged particles than charged particles [39]. The administration of Tween 80 as a surfactant in the construction of SLNs gives them an added advantage of hydrophilicity. Further, this agent increased permeability across the intestinal membrane by a high affinity between lipid particles and intestinal membrane, and it may improve bioadhesion to the gastrointestinal tract wall. Zeta potential is another important factor for stable dispersing of nanoparticles. So, having zeta potential about −5 mV produced a stable dispersion with little or no chance of aggregation [40].

According to the present results, myricitrin SLNs revealed the optimum size, zeta potential, good stability, and release. This event indicated high entrapment of myricitrin in the solid lipid matrix, preventing from the aggregate or opsonization, maintaining the effective therapeutic drug concentrations, and leads to a longer half-life. So, drug released from its SLNs was about 50% during 24 h in the present study. A slow release in initial 1 h could be explained as the slow diffusion of the drug from the inside of the solid lipid matrix. A controlled 1release pattern was depicted during 1–24 h, and it could be conducted by drug desorption from the outer surface of the SLNs and the larger specific surface of the smaller particles [41]. Since myricitrin is susceptible to the high temperature, cold homogenization has been used in our research. This method was developed to resolve various problems such as temperature-induced drug degradation, drug distribution into the aqueous phase during homogenization, and complexity of the crystallization step of the nanoemulsion [42].

Consistent with the present results, previous studies demonstrated that STZ-NA-induced diabetes is characterized by significant body weight loss due to the insulin resistance, which causes the excessive breakdown of protein as an energy source due to the inability of the body to administer glucose for producing energy [43]. An alteration in the internal organ weights may indicate toxicity or pathology occurring to them. It was revealed that pancreas weight increased in type 2 diabetic patients and STZ-induced diabetic rats [44–46]. Also, the loss of muscle weight has occurred in diabetic cases [47]. The results of myricitrin SLN administration in diabetic mice improved these alterations due to the protection from the damage of the pancreatic and muscle tissues. So, the mechanism of its action may occur through the enhancement of glycemic control and decreased catabolism of muscle's protein [48].

Lipid peroxidation has occurred in chronic diabetic condition, and MDA level increased in diabetic cases. It demonstrated that the mean activities of SOD and CAT are lower in pancreatic tissue of untreated diabetic rats. Hyperglycemia caused cellular damage through the several pathways such as increased nonenzymatic glycation and increased oxidative stress [49]. This condition elevates lipid peroxidation and reduces antioxidant defense, which leads to the increased expression of cellular damage gene products [50]. Antioxidant therapy by several compounds could prevent the lipid peroxidation [51]. Therefore, present results showed that STZ-NA-induced T2DM increased MDA and decreased CAT in the pancreas. Hence, it could be suggested that this model of diabetes may create pancreatic disorders via the increase of lipid peroxidation and H_2_O_2_. Further, the same effect in hyperglycemic exposed myotubes was observed in addition to the decrease of SOD level. SLN of myricitrin could enhance plasma antioxidant capacity in conjunction with reduced lipid peroxidation and results in maintaining redox balance in the pancreas. These effects were more evident in C2C12 cells due to the direct administration of myricitrin SLNs in the culture medium. Excessive production of antioxidant agents during oxidative stress and lipid peroxidation can exacerbate the cellular function disorders [52]. Hence, it seems hyperglycemic medium may induce more damages in muscle cells by the enhanced level of both TAC and MDA in the present study. Also, administered myricitrin at a low dose was more potent on the improvement of hyperglycemia-induced oxidative stress through increased antioxidant enzyme levels in myotubes, and this effect was more evident than metformin administration.

T2DM causes the insulin resistance that leads to hyperglycemia and reduces *β*-cell mass or secretory effectiveness [53]. The synthesis of glycogen is the main pathway for glucose uptake in the myotubes, which is regulated by hexokinase II and glucose-6-phosphate amidotransferase activity [54, 55]. In diabetic condition, the normal capacity of skeletal muscle gets impaired to synthesize of glycogen. In STZ-NA-induced diabetes model, the activation of glycogen synthase was decreased, and the activity of glycogen phosphorylase increased [56]. The Glut-4 transporter is a major site for glucose uptake which is specifically expressed in insulin-sensitive tissue such as skeletal muscles. Impaired glucose transport by a defect in insulin-mediated Glut-4 translocation induces insulin resistance [57]. Further, the impairment of glucose uptake due to the decrease Glut-4 level in skeletal muscle tissue has occurred through hyperglycemia [54, 55]. Therefore, the present study revealed that STZ-NA, as a model of type 2 diabetes, induced hyperglycemia, increased HOMA-IR, reduced HOMA-*β*, and decreased glycogen content of skeletal muscle and Glut-4 gene expression. A similar effect was evident in glycogen content and Glut-4 gene expression of untreated hyperglycemic C2C12 cells.

Flavonoids play an insulin-mimetic action, stimulate glucose uptake in peripheral tissue, and regulate the activity or expression of the rate-limiting enzymes involved in carbohydrate metabolism such as hexokinase [58]. Present results indicated that myricitrin SLN administration improved STZ-NA-induced diabetic alterations such as hyperglycemia, hyperinsulinemia, and *β*-cell's function index. However, SLN containing myricitrin consumption increased plasma level of insulin compared to untreated diabetic mice, but it could be suggested that this effect may occur through the more improved insulin secretion as a compensatory function of *β*-cell which is evident in islet diameter (as an increased *β*-cell secretory activity).

Hyperinsulinemia associated with increased insulin resistance is a demand for insulin secretion, and this event thought to reflect increased secretory capacity from the *β*-cells as a compensatory response for reducing blood glucose level during T2DM [59, 60]. Also, several studies have suggested that the T2DM process should be divided into the following three phases: hyperinsulinemia stage, prediabetes stage (impaired fasting glucose), and diabetes stage. Thus, hyperinsulinemia exists long before T2DM occurs. According to the WHO, prediabetes was typically defined as blood glucose level higher than the normal, but lower than diabetes thresholds (about 100–130 mg/dL) along with hyperinsulinemia. Hence, if physicians can provide an intervention treatment during the earlier stages of hyperinsulinemia, patients may have a better opportunity to prevent or delay the occurrence or development of T2DM. Moreover, several studies have reported that prediabetes can convert back to normoglycemia by an intervention in lifestyle and drug-based [61, 62]. So, according to the results of SLN containing myricitrin in the present study, it could be suggested that this compound increased the compensatory action of *β*-cells and improved hyperglycemia concomitant with increased plasma level of insulin and was able to restore the STZ-NA-induced diabetes to a prediabetes stage, which may be a promising new treatment for the recovery and treatment of T2DM.

Glucose uptake in skeletal muscle was mediated by mobilizing Glut-4 to the plasma membrane which stimulates insulin action. Insulin initiates glycogen synthesis in muscle via the entrance glucose into the cells. This process is the majority of whole-body glucose uptake and nonoxidative glucose metabolism [63] which was destroyed during T2DM [64]. Also, it was revealed that quercetin as a flavonoid compound improved the utilization of glucose through the increase Glut-4 gene expression and glycogen synthesis in myotubes [64]. So, the present results indicated STZ-NA-induced diabetes increased insulin resistance and decreased Glut-4 level that leads to hyperglycemia and reduced glycogen content in muscles and C2C12 cells. Moreover, myricitrin SLN administration recovered muscle's glycogen decreasing through the improved Glut-4 gene expression, insulin resistance, and glucose uptake in diabetic animals and muscle cells.

Anion superoxide level elevates during hyperglycemia and induces mitochondrial dysfunction or cellular apoptosis [65]. It was revealed that hyperglycemic condition reduced adrenal medulla and osteoblast cell viability by a decrease in MTT levels [66, 67]. The mechanism of apoptosis is regulated by Bcl-2 family of proteins including Bax and Bcl-2. Bax protein expression promotes cell death, and the increase in Bcl-2 level leads to enhancement of cell survival. One studyshowed that Bax inactivated Bcl-2 proteins via heterodimerization [68]. Bax to Bcl-2 ratio increased during the apoptosis induction and exacerbates the susceptibility of apoptotic stimuli in the hematopoietic cell [68]. One study demonstrated that hyperglycemia significantly reduces Bcl-2 and enhances Bax levels in beta-cells [69]. The administration of antioxidants such as kaempferol improves hyperglycemia-induced apoptotic changes in cells [70, 71]. Therefore, according to the previous study, the present results demonstrated that hyperglycemic medium reduced C2C12 cell viability through the decreased MTT and Bcl-2 and increased Bax gene expression and Bax to Bcl-2 ratio. Further, SLN containing myricitrin treatment improved cell viability and apoptosis via the inversion of hyperglycemic changes in a dose-dependent manner.

Beta-cell apoptosis depends on the severity and time coursing of progression T2DM, oxidative stress, and antioxidant defenses. Consistent with the results of our study, Adam et al. revealed that STZ-NA-induced T2DM could decrease islet diameter and reduce *β*-cell through increased apoptosis in them [72]. It was demonstrated that antioxidant therapy could protect *β*-cells against glucose toxicity as a beneficial treatment for T2DM [73]. Therefore, the present results indicated that myricitrin SLN utilization increased islet diameter via improved hyperglycemic induced pancreas toxicity and apoptosis.

Metformin, belonging to the biguanide, has an insulinotropic effect, reduces blood glucose level concomitant with MDA concentration, and improved the altered activities of the antioxidant enzyme [74]. It was revealed that skeletal muscle glycogen synthase activity and glycogen content were increased in diabetic metformin-treated mice [75]. Concomitant with the previous studies, and similar to the results of SLN containing myricitrin administration, metformin could improve lipid peroxidation, antioxidant defense, hyperglycemia, *β*-cell function index, glycogen content, and islet diameter or apoptosis in STZ-NA-induced diabetic mice. Also, the comparison between metformin and myricitrin SLN utilization revealed that SLN of myricitrin is more potent than metformin in the improvement of SOD level, muscle and myotube glycogen content, Glut-4 gene expression in skeletal muscle and C2C12 cells, Bcl-2 gene expression, and Bax to Bcl-2 ratio of myotubes as an apoptosis index.

## 5. Conclusion

In conclusion, SLN containing myricitrin showed antidiabetic and antioxidant effects through the recovered body and tissue weight, oxidative stress, hyperglycemia, skeletal muscle glycogen content, insulin resistance, *β*-cell's function index, Glut-4 gene expression, and pancreas apoptosis which have been altered by STZ-NA-induced T2DM. In vitro assessment revealed that SLNs of myricitrin improved the antioxidant defense, amount of glycogen, and cellular survival in myotube cells exposed to the hyperglycemic condition. Also, some of these effects were more evident in SLN-administered groups compared to the metformin group.

## Figures and Tables

**Table 1:** Effects of myricitrin SLNs on plasma level of insulin, HOMA-IR, and HOMA-*β*.

Experimental groups	Insulin (*μ*IU/mL)	HOMA-IR	HOMA-*β*
Control	17.22 ± 0.65	4.90 ± 0.21	119.69 ± 8.07
Vehicle	15.79 ± 1.97^∗^	4.38 ± 0.39^∗∗∗^	118.06 ± 6.25^∗∗∗^
Diabetes	24.31 ± 0.97^#^	13.19 ± 0.52^###^	56.12 ± 3.58^###^
Diabetes + SLN of myricitrin 1 mg/kg	30.99 ± 2.25^###$$^	10.51 ± 0.44^###^^∗^	150.963 ± 5.01^#^^∗∗∗^
Diabetes + SLN of myricitrin 3 mg/kg	33.36 ± 3.02^###^^∗^^$$^	10.95 ± 0.90^###^^∗^	172.363 ± 7.93^###^^∗∗∗^
Diabetes + SLN of myricitrin 10 mg/kg	36.02 ± 3.41^###^^∗∗^^$^	10.35 ± 1.18^###^^∗∗^	260.163 ± 15.30^###^^∗∗∗^
Diabetes + metformin 200 mg/kg	42.95 ± 2.62^###^^∗∗∗^	12.45 ± 0.30^###^	293.625 ± 10.30^###^^∗∗∗^

Data are presented as mean ± SE; *n* = 12; ^#^*p* < 0.05 and ^###^*p* < 0.001 compared with control; ^∗^*p* < 0.05, ^∗∗^*p* < 0.01, and ^∗∗∗^*p* < 0.001 compared with diabetes; ^$^*p* < 0.05 and ^$$^*p* < 0.01 compared with diabetes + metformin (one-way analysis of variance (ANOVA), followed by post hoc least significant difference (LSD) tests).

## Data Availability

The data used to support the findings of this study are available from the corresponding author upon request.
